# Recurrent primary scrotal extramammary Paget disease: a case report and literature review

**DOI:** 10.1308/rcsann.2023.0006

**Published:** 2023-04-13

**Authors:** CP Pappas, J Carroll

**Affiliations:** ^1^ The University of Queensland, Brisbane, Australia; ^2^ The Prince Charles Hospital, Chermside, Australia

**Keywords:** Extramammary Paget disease, Skin neoplasms, Perineum

## Abstract

Extramammary Paget disease (EMPD) is a rare malignant neoplasm arising in apocrine gland-rich skin, which may be classified as either of primary or secondary origin. Management of this condition is predominantly surgical, and is often characterised by lengthy diagnostic delays. Complete surgical excision is challenging, and local recurrence is common. Herein, we discuss a subtle presentation of recurrent scrotal EMPD in a 77-year-old male and review the available literature. Although relatively rare, the indistinct nature of this pathology merits special attention from treating surgeons, who are frequently responsible for initial management and follow-up. The risk of distant metastasis and concomitant prognostic implications necessitate a high clinical index of suspicion, and low threshold for definitive biopsy in similar cases.

## Case history

**Figure 1:**
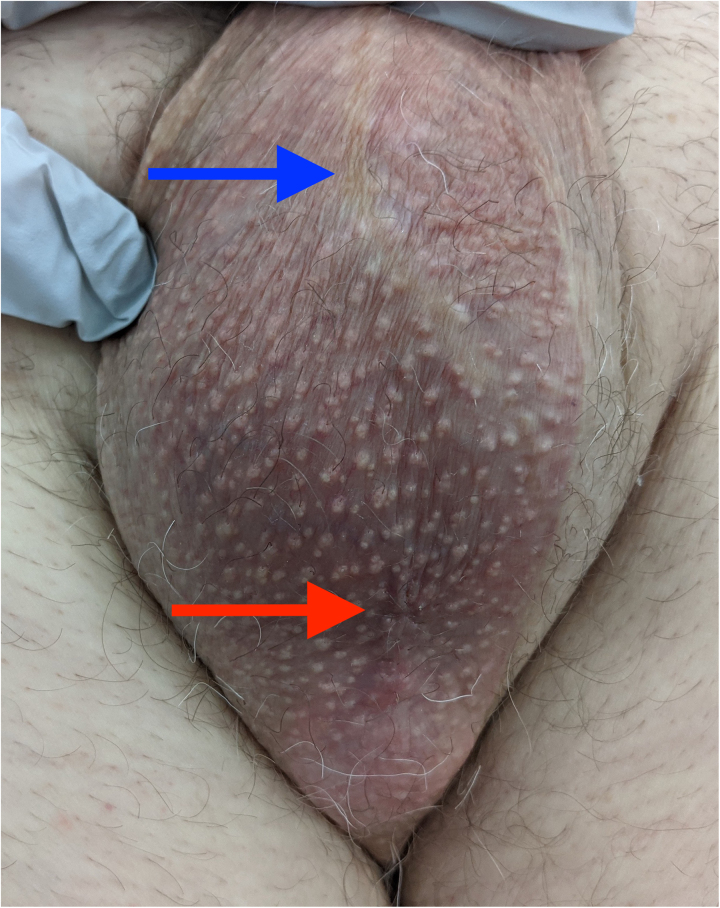
Preoperative image of the patient’s scrotum, demonstrating the virtually indistinct nature of the lesion. The scar from the previous excision superiorly (blue arrow) and the incisional biopsy scar inferiorly (red arrow) are visible.

A 77-year-old male presented to our institution with a 4-week history of a mildly pruritic scrotal rash. Topical miconazole antifungal therapy was previously trialled by his primary care physician with no improvement. Importantly, the patient’s background included known primary scrotal extramammary Paget disease (EMPD), managed surgically 5 years previously with apparent cure. On examination, the scrotum displayed a subtle, non-blanching, violaceous appearance limited by the scar of the previous excision, resembling a benign fungal or inflammatory dermatosis (Figure 1). There was no evidence of ulceration or nodules that might indicate an advanced malignant process,^1^ and the lesion was virtually unappreciable.

**Figure 2:**
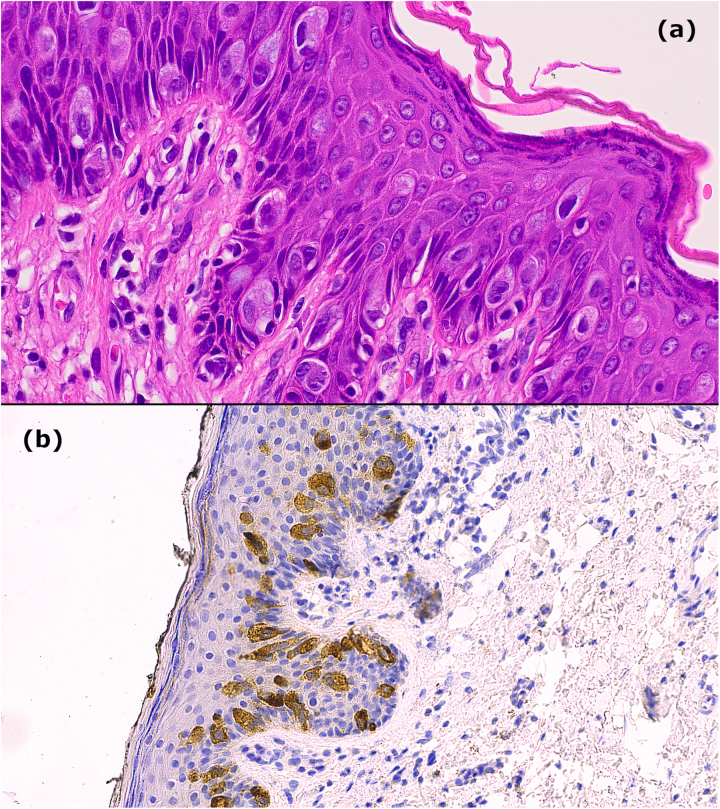
(a) Haematoxylin and eosin staining shows large, atypical Paget cells with abundant cytoplasm and vesicular nucleoli, with adnexal involvement and chronic inflammatory dermal infiltrate. (b) Carcinoembryonic Antigen (CEA) staining in atypical Paget cells. Immunohistochemistry also revealed positive staining with Cytokeratin 7 (CK7), Cytokeratin 20 (CK20) and Epithelial Membrane Antigen (EMA). There is no evidence of dermal invasion.

Given the patient’s history, incisional biopsy was performed confirming EMPD with no dermal invasion (Figure 2). Secondary causes had been excluded previously through systematic workup. We subsequently performed a wide local excision of scrotal skin with primary closure. Owing to the indistinct nature of the lesion, a 1cm margin was taken. Unfortunately, an involved margin at 6 o’clock necessitated re-excision. Postoperatively, the role of adjuvant radiation therapy was discussed but ultimately not felt to be necessary given treatment-associated morbidity and the patient’s age.

The patient is engaged with regular postoperative follow-up and has displayed no recurrence of his symptoms to date.

## Discussion

The available literature on EMPD reflects the condition’s rarity, with an estimated incidence of 0.1 to 2.4 per 1,000,000 patient years, most commonly in patients aged over 50 years.^1^ EMPD demonstrates a female preponderance in Caucasian populations (male-to-female ratio 1:1.2),^2^ in whom disease typically presents as vulval lesions; penoscrotal disease is relatively rare accounting for only 14% of cases.^1,3^ Unknown genetic or sociocultural variables also appear to play a role, with some studies suggesting a fourfold risk of disease among Asian and Pacific Islander populations, in whom the male-to-female ratio approaches 1:1.^1^ Ultimately, the risk factors for EMPD remain largely unknown.

Of relevance is the distinction between primary and secondary EMPD, where primary disease represents in situ intraepidermal neoplasia via malignant transformation of pluripotent keratinocyte stem cells or apocrine gland duct cells.^1^ Secondary disease is comparatively less frequent, occurring in 7%–40% of cases, and represents epidermotropic spread from a distant primary adenocarcinoma.^1^ Importantly, histopathological analysis cannot reliably differentiate between primary and secondary disease, and so secondary causes of EMPD must always be excluded.

Wide local excision is the most common treatment modality for scrotal EMPD. However, recurrence rates reported in the literature are high at 30% to 60%, with challenges posed by clinically ill-defined margins, and multifocal disease.^3^ Alternative surgical options include Moh’s micrographic surgery, although reported recurrence rates of 11% remain high compared with Moh’s treatment of non-melanomatous skin cancers.^4^ Although there is no consensus regarding appropriate surgical margins, with practice ranging from 1 to 5cm, previous studies suggest wide margins do not appear to reduce recurrence rates.^4^ Non-surgical treatments with some evidence of efficacy include Imiquimod and photodynamic therapy,^4^ and local radiotherapy,^3^ but no single regimen has been defined. There is no defined follow-up rationale, although clinical review is the foundation, with a low threshold for repeat biopsy as seen in this case.

Although primary disease exhibits indolent growth and retains a favourable prognosis compared with secondary disease, dermal invasion is associated with regional lymph node involvement and subsequent metastatic potential carrying a reported 5-year survival rate as low as 7%.^5^

This case highlights the key challenges in managing EMPD. First, the disease is rare, and lesions often present as indistinct erythematous rashes with non-specific symptoms, such as mild pruritis. Accordingly, the diagnosis is not always apparent, as seen in our case where initial primary care treatment was for a fungal rash despite known scrotal EMPD. In other cases, this may contribute to a delayed diagnosis, carrying a poor prognosis. Moreover, the multifocal nature of EMPD and the difficulty encountered in visualising lesion extent intraoperatively make the surgical management of EMPD challenging, as reflected by the presence of recurrent disease and positive margins in our case.

## Conclusion

Ultimately, this case underscores the need for treating surgeons to maintain a high index of suspicion when encountering skin lesions that have not responded to topical therapies, particularly in high-risk areas including the scrotum and perineum. A low threshold for definitive biopsy will minimise diagnostic delays and the prognostic implications thereof.

## Patient consent

Patients have given informed consent to the publication of images and data.
